# A high-throughput gut-on-chip platform to study the epithelial responses to enterotoxins

**DOI:** 10.1038/s41598-024-56520-5

**Published:** 2024-03-09

**Authors:** Moran Morelli, Marta Cabezuelo Rodríguez, Karla Queiroz

**Affiliations:** Mimetas, Oegstgeest, The Netherlands

**Keywords:** Toxicology, Mechanisms of disease

## Abstract

Enterotoxins are a type of toxins that primarily affect the intestines. Understanding their harmful effects is essential for food safety and medical research. Current methods lack high-throughput, robust, and translatable models capable of characterizing toxin-specific epithelial damage. Pressing concerns regarding enterotoxin contamination of foods and emerging interest in clinical applications of enterotoxins emphasize the need for new platforms. Here, we demonstrate how Caco-2 tubules can be used to study the effect of enterotoxins on the human intestinal epithelium, reflecting toxins’ distinct pathogenic mechanisms. After exposure of the model to toxins nigericin, ochratoxin A, patulin and melittin, we observed dose-dependent reductions in barrier permeability as measured by TEER, which were detected with higher sensitivity than previous studies using conventional models. Combination of LDH release assays and DRAQ7 staining allowed comprehensive evaluation of toxin cytotoxicity, which was only observed after exposure to melittin and ochratoxin A. Furthermore, the study of actin cytoskeleton allowed to assess toxin-induced changes in cell morphology, which were only caused by nigericin. Altogether, our study highlights the potential of our Caco-2 tubular model in becoming a multi-parametric and high-throughput tool to bridge the gap between current enterotoxin research and translatable in vivo models of the human intestinal epithelium.

## Introduction

The gastrointestinal mucosa separates the internal environment from the external lumen^1^. It conforms a semipermeable barrier that integrates the commensal microbiota, a mucus layer, the intestinal epithelium, and resident immune cells. Among these components, the intestinal epithelium is the main physical structure regulating barrier function. As a continuous, polarized, single layer of cells – of which enterocytes are most abundant, it allows nutrient absorption and simultaneously limits the entry of harmful molecules and pathogens. Substances absorbed into the mucosa can passively diffuse through the epithelium, be carried transcellularly via selective cell transporters, or pass across the space between adjacent cells in a paracellular manner. This last mode of substance uptake is regulated by tight junctions (TJs) that seal the apical intercellular space^2^.

Enterotoxins define a wide range of toxins secreted by various organisms that target the intestinal epithelium, leading to increased intestinal permeability and epithelial damage. These pathogenic mechanisms are heterogeneous and still under investigation. Clostridium perfringens enterotoxin CPE and honeybee venom melittin directly alter barrier structure by configuring transmembrane pores that enhance transcellular permeability^3,4^. Other toxins disrupt TJs by promoting degradation of TJ proteins or disorganizing the actin cytoskeleton, resulting in increased paracellular permeability, such as *Clostridium difficile*’s toxin A or mycotoxin patulin^5^. Induction of apoptosis or necrosis by mycotoxins ochratoxin A and deoxynivalenol lead to cell death and loss of barrier integrity. Consequently, tissue damage and the entry of exogenous molecules into the mucosa trigger a pro-inflammatory response in the epithelium, causing the release of inflammatory mediators such as IL-1B, IL-8 and TNF-α^6^. This host defense response also appears to be toxin-specific: patulin exerts distinct immunosuppressive effects^7^, and *Candida albicans*’ candidalysin triggers the release of pathogen-specific alarmins and antimicrobial peptides^8^.

Enterotoxin intoxication results in acute varying gastrointestinal symptoms including nausea, vomiting and diarrhea, which manifest within hours or days after ingestion^9,10^. Recent studies have uncovered that, in addition to epithelial damage, several mycotoxins commonly found in foods also have long-term carcinogenic, mutagenic, hepatotoxic, and nephrotoxic effects^11–13^. Moreover, loss of barrier integrity is a common feature of inflammatory bowel disease, celiac disease, and ulcerative colitis^4^, as well as non-gastrointestinal diseases such as type 2 diabetes^14^ multiple sclerosis^15^ and Parkinson’s disease^16^. The sustained state of low-grade inflammation associated with chronic toxin exposure could therefore contribute to the development of these inflammatory diseases. Furthermore, co-occurrence of mycotoxins and bacterial toxins is now a matter of concern for food safety authorities, as this may lead to additive or synergic cytotoxic effects^17,18^. Thus, considering the severe pathological implications tied to enterotoxin exposure, precise evaluation of enterotoxin-specific epithelial toxicity is needed to establish safe enterotoxin exposure thresholds.

Interest in enterotoxin research has shifted beyond food safety concerns. Bacterial toxins zonula occludens toxin (ZOT) and clostridium perfringens enterotoxin (CPE) show potential as permeability enhancers to improve intestinal absorption of oral drugs^19^. Seeing the vast array of enterotoxins that increase barrier permeability, research would benefit from a platform that enables rapid enterotoxin screening. Antibodies, small molecules, and inhibitors targeting pore-forming toxins are being developed as adjuvants to conventional therapy against infection. Recently, Ernst et al. used chloroquine to inhibit the cytotoxic effects of the pore-forming toxin CDT from the intestinal pathogen Clostridioides difficile^20^. Additionally, dietary components are being tested for their ability to recover the intestinal barrier after enterotoxin exposure, such is the case for galacto-oligosaccharides, which alleviated loss of barrier function after exposure to mycotoxin deoxynivalenol as reported by Akbari et al.^21^.

However, the use of animal in vivo models and two-dimensional in vitro models in most enterotoxin clinical and food safety studies limits the model-to-human extrapolation of the findings. In vivo studies provide the highest level of physiological complexity to assess enterotoxin toxicity. However, low throughput, interspecies variability and ethical dilemmas surrounding animal experimentation highlight the need for robust, complex, and clinically relevant alternative methodologies^22^. Current in vitro intestinal models have failed to mimic the complexity of the intestinal barrier while remaining a cost-effective, non-laborious and high-throughput approach. Transwell models, though suitable for fast toxin screening, fail to replicate the perfused and tubular structure of the intestine and lack interaction with the extracellular matrix (ECM)^23^. This interaction, crucial for cell differentiation, morphogenesis, and proliferation, has successfully been modelled by three-dimensional, organoid-based, and microfluidic organ-on-a-chip models, although they often rely on artificial barriers to support the ECM^24,25^. Some even incorporate media perfusion circuits that allow dynamic media flow and induce shear stress, which is also key to establish physiological intestinal cell morphology and polarization^26^. However, the reliance on pump systems in these models makes them unsuitable for large-scale compound screening^25^.

The OrganoPlate is a membrane-free, high-throughput organ-on-a-chip platform that integrates up to 64 parallel microfluidic channels, allowing the culture of cells in direct contact with the ECM and continuous media perfusion^27,28^. As shown by Trietsch et al., culture of adenocarcinoma-derived Caco-2 cells in the OrganoPlate more closely resembles the differentiation, polarization, and gene expression profiles of the intestine than *Transwell* systems^27^. Further studies also showed how the platform can effectively mimic intestinal inflammatory disease conditions and can be used to assess intestinal drug permeability^29,30^. More recently, Pöschl et al. reported the use of the platform to examine the effects of deoxynivalenol on barrier permeability, being the first to study enterotoxin toxicity using a high-throughput in vitro model with flow^31^. Together, these studies showcase the emerging potential of Caco-2 tubules as a model for toxicological assessment of enterotoxins.

Here, we aimed to design and optimize a gut-on-a-chip model with Caco-2 tubules to study the effects on permeability, morphology and inflammation induced by microbial enterotoxins on the intestinal epithelium. To do so, we selected four toxin candidates with various pathogenic mechanisms – nigericin, patulin, ochratoxin A and melittin – and assessed intestinal barrier and cellular damage. As a result, we report how our caco-2 tubular model can be used as a robust, high-throughput, in vitro platform to study enterotoxin-specific toxicity in the intestinal epithelium.

## Materials and methods

### OrganoPlate culture

**Figure 1:**
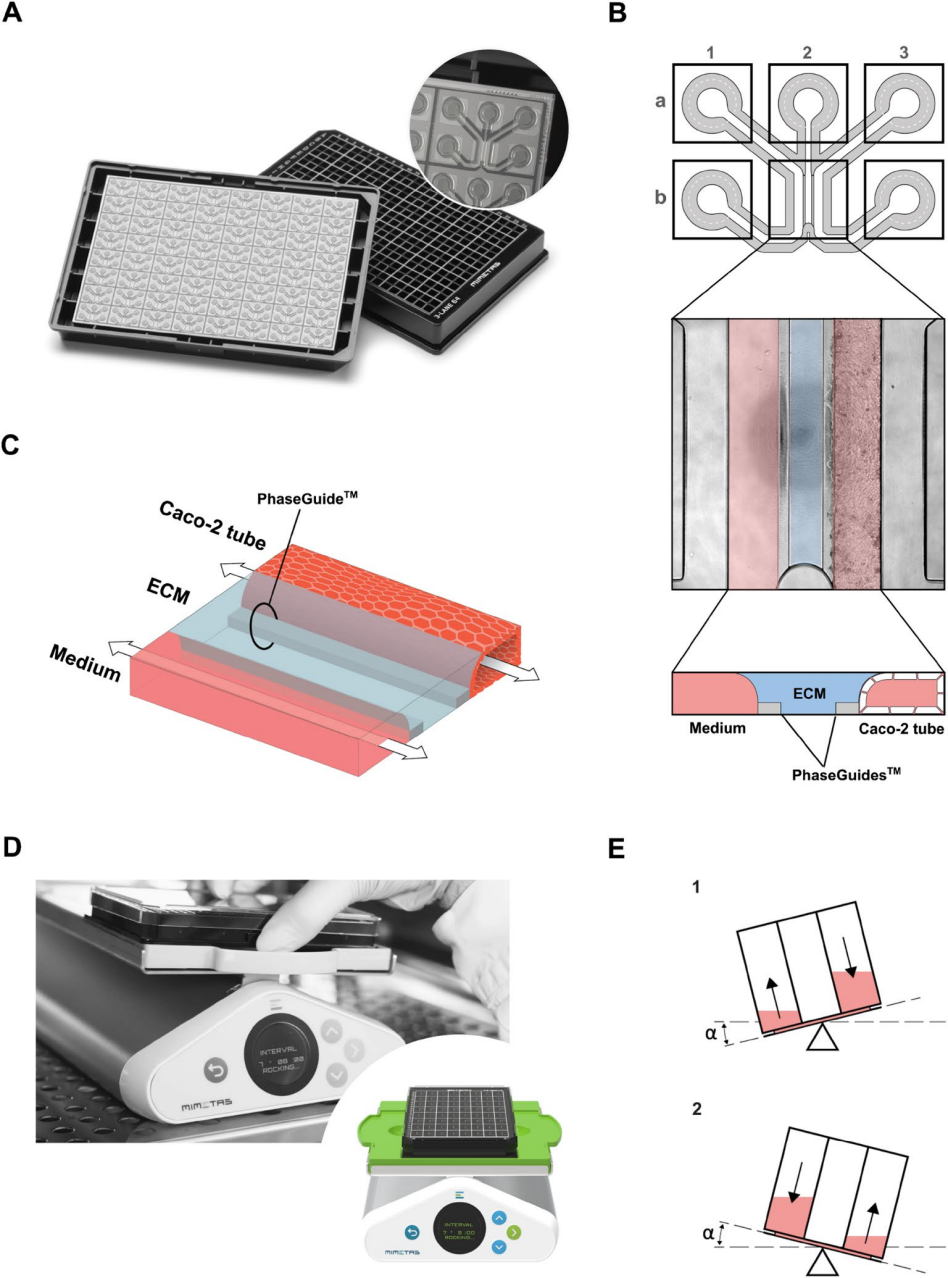
The OrganoPlate and OrganoTEER allow perfused culturing of Caco-2 cell tubules. (**A**) Photograph of the bottom and top of the OrganoPlate, showing 64 microfluidic channel chips embedded in a standard 384-well microtiter plate and a zoomed-in view of a single chip. (**B**) Schematic picture depicting the structure of a single microfluidic chip, which consists of three channels: the left channel (1), middle channel (2) and right channel (3), which are accessible via inlets (1a, 2a, 3a) and outlets (1b, 3b). Squares represent the access wells of the 384-well plate. Imaging and direct observation of the culture is possible via the observation window (2b). A top view phase-contrast image of the observation window and a transversal view schematic diagram of the observation window is provided. These pictures show the right channel where the Caco-2 cells form the epithelial tubule, the middle channel seeded with extracellular matrix (ECM) and the left channel filled with medium. The right and left channels are separated from the middle channel thanks to the PhaseGuides, which allow barrier-free channel separation. (**C**) Diagram picturing the tridimensional structure of a perfused chip, indicating how the Caco-2 tubule channel and the medium channel undergo bidirectional medium flow. (**D**) Photographs of the OrganoFlow rocker device, on top of which OrganoPlates are placed to induce medium flow. (**E**) Transversal view of a channel that shows how bidirectional medium flow is generated by continuous angular tilting of the OrganoPlate by the OrganoFlow rocker.

The OrganoPlate is a 3D culture plate with 64 (3-lane configuration) microfluidic tissue model chips built into a standard 384 well plate (Fig. 1A). Extracellular matrix (ECM) gels are patterned in microfluidic channels using PhaseGuides™ (Fig. 1B,C). After gelation of the ECM, cells are seeded in the perfusion channel and the plate is placed on the MIMETAS OrganoFlow rocker (Mimetas BV, The Netherlands) to form perfused intestinal microtubules (Fig. 1D,E)^27^. Seeding of ECM and cells was described by Trietsch and colleagues^27^. In this study, we used OrganoReady Colon Caco-2 (Mimetas BV, The Netherlands), further referred to as Caco-2 tubules, comprising 64 ready-to-use caco-2 gut tubules seeded against collagen I, allowing to perform assays right away.

### Enterotoxin exposure

Caco-2 tubules were exposed to toxins nigericin (Sigma-Aldrich, SML1779), patulin (Sigma-Aldrich, P1639), ochratoxin A (Sigma-Aldrich, 494,128) and melittin (Sigma-Aldrich M4171) after 7–8 days of culture to ensure complete cell confluency and intestinal barrier formation. Cells were serum-starved overnight before enterotoxin exposure to avoid possible interactions with serum factors; this was performed by refreshing all inlets and outlets from the right and left channels with serum-free EMEM medium. Before exposure, transepithelial electrical resistance (TEER) measurements and phase-contrast images were recorded from all chips to establish individual baseline permeability conditions of the tubules (t = 0). After this, apical media from the right channel inlets and outlets was substituted with the different enterotoxin medium dilutions.

Toxins were diluted in sterile milliQ or DMSO in a 1:3 decreasing serial manner: concentrations of nigericin used ranged from 0.007 to 5 μg/mL, concentrations of patulin from 0.34 to 250 μg/mL, concentrations of ochratoxin A ranged from 0.0013 to 1 μg/mL,and concentrations of melittin ranged from 0.02 to 50 μg/mL. These toxin dilutions were then adjusted to a maximum 0.01% or 5% dilution in serum-free EMEM, depending on whether the vehicle was DMSO or milliQ respectively, to avoid possible cytotoxic effects caused by the vehicle^32^. An adjusted dilution of vehicle containing enterotoxin was always included as a negative control. For the NF-kB experiments, an inflammatory cocktail dilution consisting of IL-1B (Immunotools, 11,340,013) and TNF-α (Immunotools, 11,343,015) at a concentration of 100 ng/mL was included as positive control. Immediately after enterotoxin exposure, plates were placed on the OrganoFlow rocker inside a humidified incubator at 37 °C and 5% CO_2_ during the indicated exposure time.

### Transepithelial electrical resistance (TEER)

**Figure 2:**
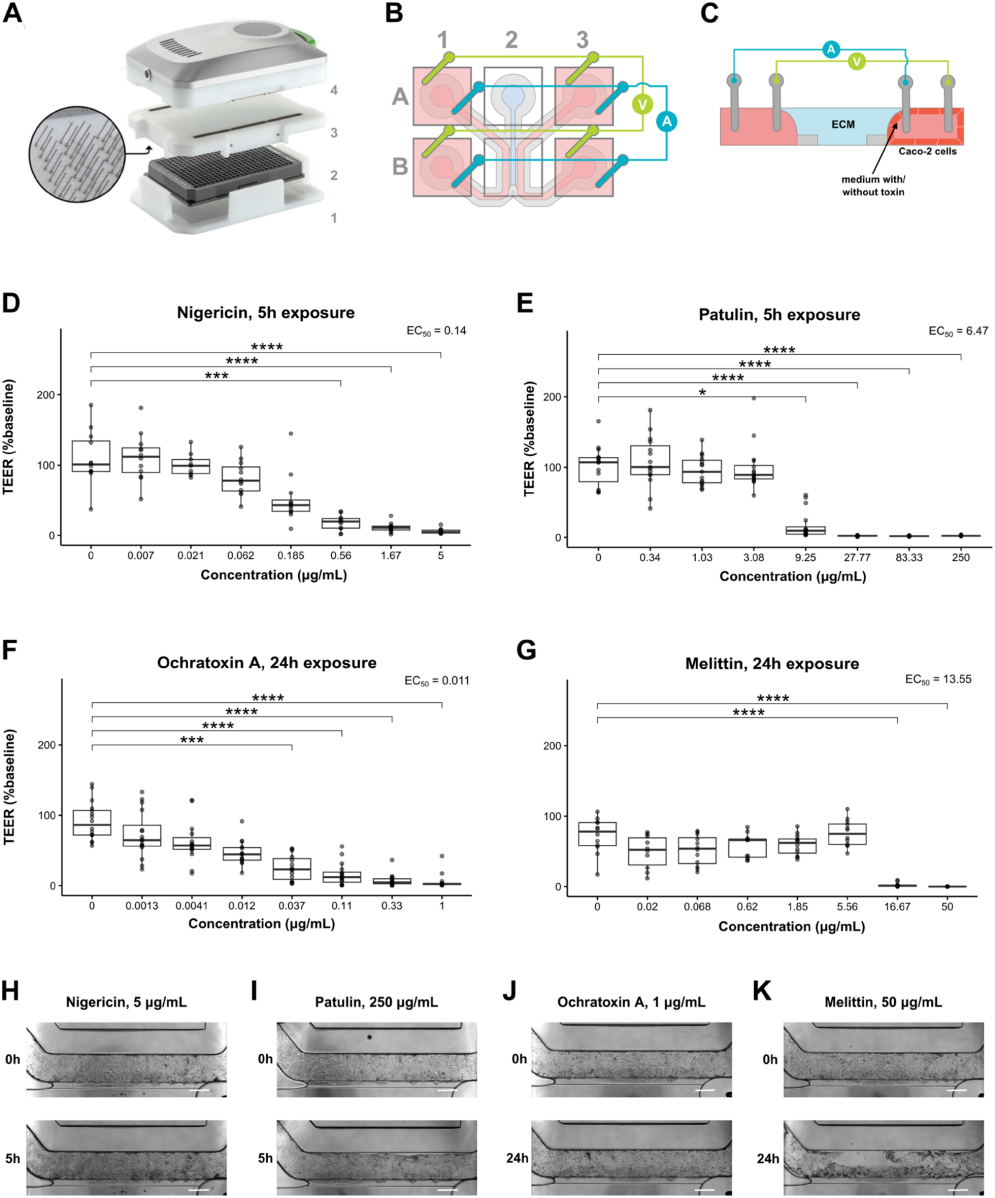
Exposure to enterotoxins leads to dose-dependent loss of barrier integrity. (**A**) Photograph of the OrganoTEER setup: the plate holder (1) holds the OrganoPlate (2) in which the electrode board (3) is positioned and connected to the measuring device (4). An expanded view of the electrodes is shown. (**B**) Schematic diagram showing the electrode positioning in a single OrganoPlate chip. Two electrode pairs are inserted into the chip wells. Current-carrying electrodes (blue) impose an AC voltage across the chip and voltage-sensing electrodes (green) measure the resulting current. (**C**) Schematic diagram showing a transversal view of the OrganoPlate chip and the electrode pair positioning across the medium channel and the Caco-2 tube channel containing the medium dilution with/without enterotoxin. (**D**–**G**) Dose-dependent decrease in TEER after a 5 h exposure to nigericin (**D**) and patulin (**E**), and a 24 h exposure to ochratoxin A (**F**) and melittin (**G**). Caco-2 tubules were incubated with various concentrations of enterotoxin diluted in medium. TEER values were normalized to baseline TEER values taken before exposure (t = 0). EC50 values obtained after fitting the TEER data with a dose–response regression model are indicated. Three independent experiments (N = 3) were performed and 2–4 technical replicates (n = 2–4) were included per enterotoxin concentration tested. Significance was determined using Wallis test followed by Dunn’s post hoc test, where **** *p* < 0.0001; *** *p* < 0.001; ** *p* < 0.01; * *p* < 0.05. **H–K** Phase contrast images of the tubules before (t = 0) after a 5 h exposure to the highest concentrations of nigericin (**H**) and patulin (**I**), and a 24 h exposure to ochratoxin A (**J**) and melittin (**K**). Pictures are representative of three independent experiments (N = 3) with 2–4 technical replicates (n = 2–4) per enterotoxin concentration tested. Scale bar in white = 300 µm.

To determine the specific enterotoxin effects on barrier permeability of Caco-2 tubules, TEER of the OrganoPlate was measured after toxin exposure. This was performed using an automated multichannel impedance spectrometer (OrganoTEER, Mimetas BV) and 3-lane-OrganoPlate-fitting electrode boards as described by Nicolas et al.^33^ (Fig. 2A) OrganoPlates were equilibrated at room temperature 30–40 min before TEER measurement by placing them on a steady surface outside the incubator, to eliminate potential effects caused by differences in temperature and flow. Point impedance TEER measurements, given by the OrganoTEER software in Ω*cm^2^, were normalized to baseline TEER at t = 0, unless indicated otherwise, to account for possible permeability variations among tubules due to differences in vehicle, timing, and shear stress effects.

### Caco-2 tubule visualization and imaging

Images were captured using the ImageXpress Micro XLS (Molecular Devices) and Micro XLS-C High Content Imaging Systems (Molecular Devices) and processed using Fiji^34^ to enhance contrast and improve visualization. To monitor the integrity of the Caco-2 tubules, phase-contrast images were recorded before and after exposure to enterotoxins. This was routinely performed immediately after measurement of baseline TEER and after the required toxin exposure time. Fixed and stained OrganoPlates were stored at 4 °C until imaging and equilibrated at room temperature at least 30 min before imaging. Maximum intensity projection images were saved as TIFF files after confocal imaging of stained cells.

### Immunohistochemistry

For visual characterization of the enterotoxin effect on actin cytoskeleton, cell permeability and NF-kB activation, cells were directly fixated and stained on the OrganoPlate based on the protocol described by Trietsch et al*.*^27^.

#### Fixation

Immediately after the required exposure time, Caco-2 tubules were fixed with 3.7% formaldehyde (Sigma, 252,549) in HBSS with Calcium and Magnesium (Thermo Scientific, 14,025,092) for 15 min, washed twice with phosphate-buffered saline (PBS; Gibco, 70,013,065) for 5 min and then stored with 50μL PBS per well at 4 °C until staining.

#### Actin and nuclear staining

Caco-2 cells were permeabilized with 0.03% Triton X-100 (Sigma, T8787) in PBS for 10 min and washed twice with 4% FBS in PBS solution. Actin and nuclear stainings were performed using the direct stains ActinGreen 488 ReadyProbes Reagent (Invitrogen, R37110) and NucBlue Fixed Cell ReadyProbes Reagent (Invitrogen, R37606). Direct staining was performed following the manufacturer’s instructions and under constant flow.

#### DRAQ7 staining

Before fixation, the adjusted medium dilutions of the tubule inlets and outlets was substituted with 20μL of DRAQ7 dye (Biostatus, DR71000) diluted 1:100 in serum-free EMEM, and cells were incubated during 30 min under continuous perfusion inside the incubator. OrganoPlates were then fixated and stained following the previously explained protocol.

#### NF-kB p65 staining

Caco-2 cells were permeabilized with 0.03% Triton X-100 (Sigma, T8787) in PBS for 10 min, washed twice with 4% FBS in PBS solution and incubated with blocking solution (2% FCS, 2% BSA, 0.1% Tween-20 (Sigma, P9616) for 45 min. Cells were then stained overnight with 1:400 NF-kB p65 (D14E12) XP primary antibody dilution (Cell Signaling Technologies, 8242), as validated by the manufacturer, at 37 °C on the OrganoFlow rocker. After washing three times with 4% FBS in PBS, cells were incubated with a 1:250 goat anti-rabbit AFTM 647 secondary antibody dilution (Invitrogen, A-21244) as indicated by the manufacturer, for 30 min on the OrganoFlow rocker at room temperature. Then cells were washed again three times with 4% FBS in PBS and 50μL of PBS were added to all wells before storing the stained OrganoPlate at 4 °C.

### Lactate dehydrogenase (LDH) release analysis

LDH activity was determined in the apical tubule media after enterotoxin exposure using the LDH-Glo Cytotoxicity Assay Kit (Promega, J2381). Medium from the right upper and bottom channels was collected and diluted 1:100 in LDH storage buffer (200mM Tris–HCl (Sigma-Aldrich, T2194), 10% glycerol (Sigma, G5516), 1% BSA (Sigma-Aldrich, A2153) and then processed following the manufacturer’s instructions. Those cells incubated with no enterotoxin-containing media were used as negative controls. White half-volume 96-well plates (Greiner, 675,075) were used to perform the assay. Luminescence was measured using the Spark Cyto plate reader (Tecan Life Sciences) using an integration time of 80 ms.

### Actin disorganization and DRAQ7-positive cell viability quantification

Open-source cell image analysis software CellProfiler^35^ (version 4.2.5) was used to process visual immunofluorescence images. An image analysis pipeline was designed to quantify the number and area of actin clumps and the number of cells, by segmentation of the actin clumps and nuclei and measurement of the total actin clump area. This pipeline was used to process the TIFF files that captured actin staining or nuclei staining, correspondingly. Parallelly, another pipeline was designed to process the TIFF files capturing DRAQ7 and nuclei staining, that allowed segmentation of DRAQ7-positive cells and nuclei, to quantify the percentage of DRAQ7-positive (DRAQ7 +) cells. A visual explanation of these CellProfiler pipelines is provided in Figs. 4A and 5A.

### Visual data quantification and data analysis

Open-source cell image analysis software CellProfiler was used to quantify visual data from actin and DRAQ7 immunofluorescence TIFF image read-outs. Visual explanation of the pipelines can be found in Figs. 4A and 5A.

Descriptive and statistical analysis were performed using open-source software R Studio v2023.03.0 and packages rstatix (v0.7.2, A. Kassambara et al.), drc (v3.0–1, C. Ritz and J.C. Strebig) and DescTools (v.99.48, Signorel et al*.*).

Experiments were performed three times in an independent manner (N = 3) unless indicated otherwise, using a total of 2–4 technical replicates per enterotoxin concentration and treatment (n = 2–4). Data was always normalized to the mean measurement of the non-exposed vehicle control chips. The dose–response models and resulting EC50 values were obtained after fitting the TEER dose–response data analysis using RStudio package drc.

Normal distribution and equality of variances were evaluated, respectively, using the Shaphiro-Wilk normality test and the Levene test for the equality of variances. Differences among groups were compared using one-way ANOVA or Kruskal–Wallis tests, followed by post-hoc Dunnett’s or Dunn’s tests to obtain pairwise comparisons between the effect of exposure to the various enterotoxin concentrations and exposure to the vehicle control.

## Results

### Toxins increase barrier permeability in a dose-dependent manner.

First, to evaluate the dose-dependent effects exerted by the different enterotoxins on the Caco-2 barrier permeability, TEER was measured using the OrganoTEER device (Fig. 2A–C). As expected, these responses were time and concentration-specific for every toxin tested (Fig. 2D–G). A preliminary study allowed us to pinpoint t = 5 h and t = 24 h as the two main exposure timepoints after which a complete dose–response relationship could be observed following toxin exposure (Supplementary Figure S1). EC50 values, which correspond to the dose at which TEER is decreased by 50%, can be calculated after fitting the dose–response TEER data with a dose–response regression model (Supplementary Figure S2). After a 5 h exposure, nigericin’s EC50 value (EC50 = 0.14 μg/mL) is lower than that of patulin (EC50 = 6.47 μg/mL), and after a 24 h exposure, ochratoxin A’s (EC50 = 0.011 μg/mL) is lower than that of melittin (EC50 = 13.55 μg/mL). This indicates that, respectively, the toxin potencies of ochratoxin A and nigericin are higher than those of patulin and melittin. Visual analysis of the fitted dose–response TEER data reveals additional differences in toxicity behavior, which can be evaluated in terms of the curve slope: while mycotoxins nigericin and ochratoxin A show shallow slopes, which are indicative of high toxicity at low doses, mycotoxin patulin and melittin display steeper slopes, indicating higher toxicity at high doses. These results also show visual differences in non-observed effect level (NOEL) values, with patulin and melittin exerting a significant drop in TEER at lower concentrations than the rest of the toxins tested. Examination of phase-contrast images of the tubules corroborates the differences observed in toxicity behavior. While exposure to the highest concentrations of nigericin and ochratoxin A did not lead to remarkable change in morphology, exposure to the highest concentrations of patulin often led to detachment of the tubule from the microfluidic channel, and exposure to the highest concentrations of melittin completely detached the tubule from the channel (Fig. 2H–K).

### Melittin is a cytotoxic toxin that causes LDH release

**Figure 3:**
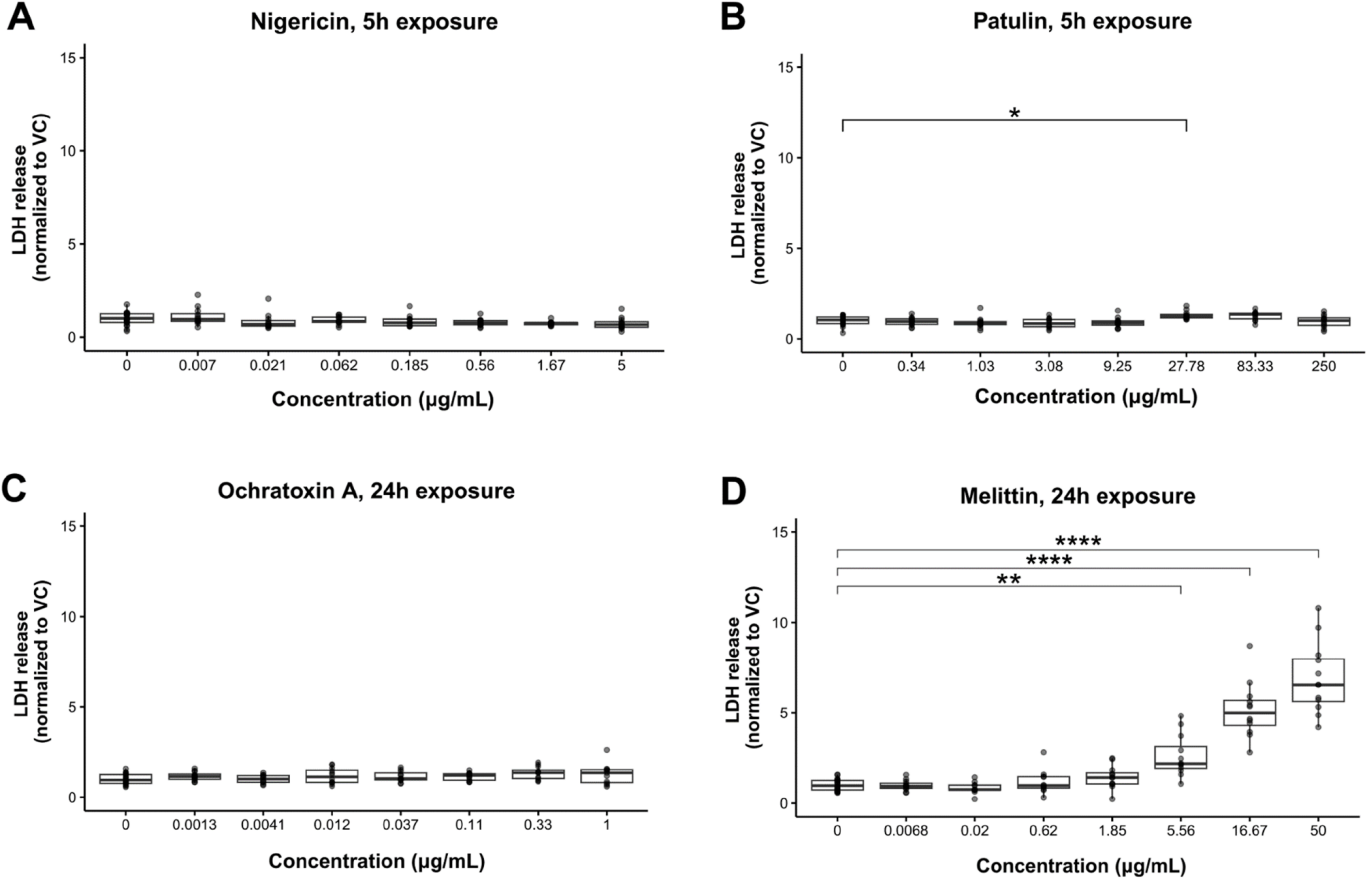
Only Melittin showed dose-dependent increase in LDH. LDH release was quantified in the Caco-2 channel medium after a 5 h exposure to nigericin (**A**) and patulin (**B**), and a 24 h exposure to ochratoxin A (**C**) and melittin (**D**). LDH concentrations in enterotoxin-exposed tubules were normalized with those of the vehicle control. Three independent experiments (N = 3) were performed and 2–4 technical replicates (n = 2–4) were included per enterotoxin concentration tested. Significance was determined using Kruskal–Wallis or ANOVA test followed by Dunn’s or Dunnett’s post hoc tests respectively, where *** *p* < 0.001; ** *p* < 0.01; * *p* < 0.05.

To evaluate the cellular toxicity, we measured the LDH release in the apical cell culture media of the Caco-2 tubules after exposure to the toxins. Figure 3 shows toxin-specific effects in LDH release. Exposure to melittin during 24 h showed a significant dose-dependent increase in LDH release starting at 5.56 μg/mL, when compared with exposure to vehicle (Fig. 3D). In contrast, exposure to neither nigericin (Fig. 3A), patulin (Fig. 3B) nor ochratoxin A (Fig. 3C) led to significant increases in LDH release.

### Melittin and ochratoxin A show increased permeability to DRAQ7 Dye

**Figure 4:**
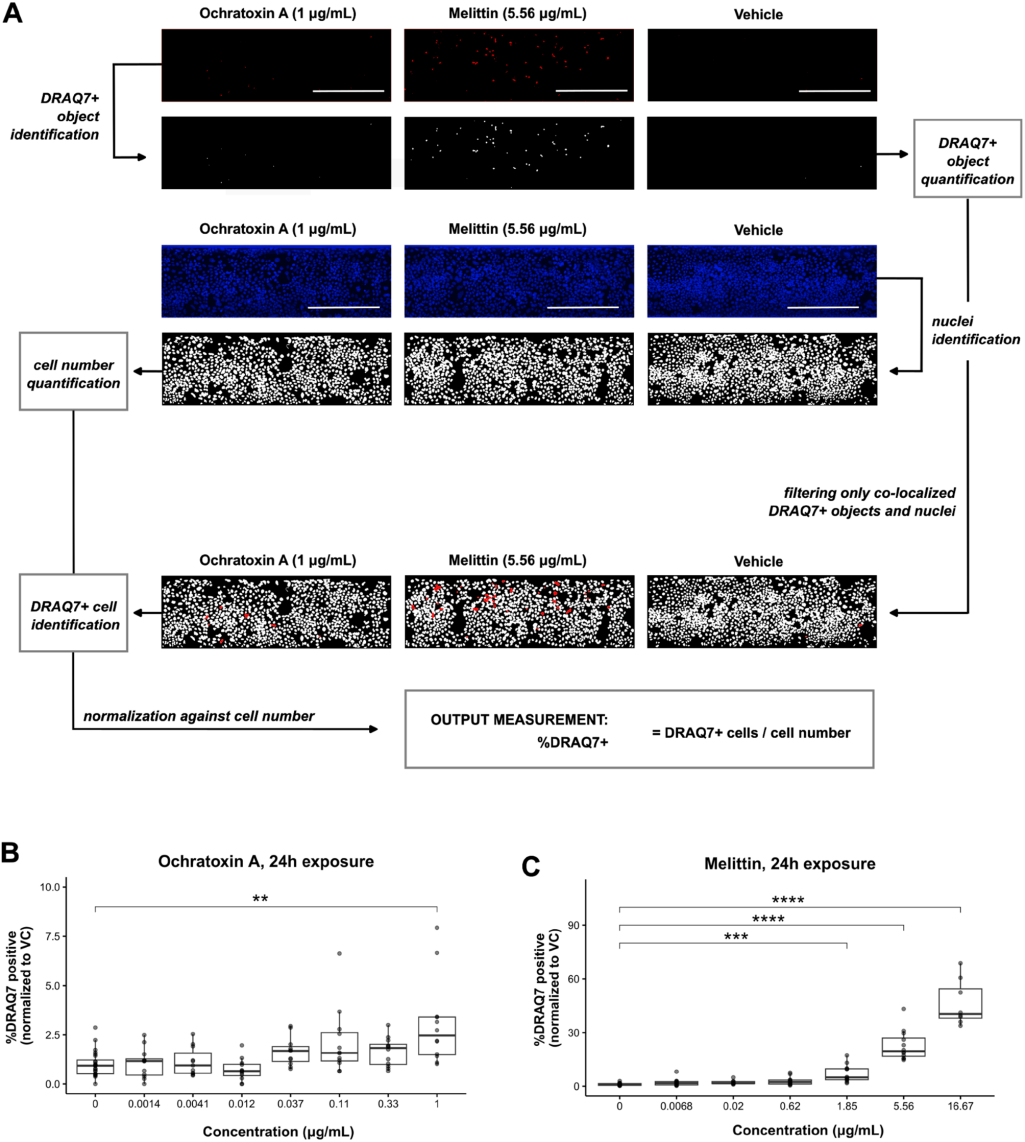
DRAQ7 staining allows assessment of the effect on cytotoxicity and cell permeability induced by ochratoxin A and melittin. Caco-2 tubules exposed to ochratoxin A and melittin during 24 h were stained with DRAQ7 nuclear dye, which only penetrates permeabilized or dead cells. Nuclei were stained with NucBlue Fixed Cell ReadyProbes Reagent. (**A**) A CellProfiler pipeline was designed to identify and quantify DRAQ-positive (DRAQ +) objects and nuclei from confocal images. DRAQ7 + objects were filtered to only consider those overlapping nuclear objects, which were identified as DRAQ7 + cells. The number of DRAQ7 + cells was normalized against the cell number, obtaining the output measurement %DRAQ7 + , which was considered a measure of enterotoxin cytotoxicity and cell permeability. Pictures are representative of three independent experiments (N = 3) with 2–4 technical replicates (n = 2–4) per enterotoxin concentration tested. Scale bar in white = 300 µm. (**B**,**C**) Dose-response increase in %DRAQ7 + cells after a 24 h exposure to ochratoxin A (**B**) and melittin (**C**). Three independent experiments (N = 3) were performed and 2-4 technical replicates (n = 2–4) were included per enterotoxin concentration tested. Significance was determined using Kruskal-Wallis test followed by Dunn’s post hoc test, where **** *p* < 0.0001; *** *p* < 0.001; ** *p* < 0.01; * *p* < 0.05.

As an additional readout to assess enterotoxin-induced cytotoxicity and transcellular permeability, we stained the exposed Caco-2 tubules with DRAQ7. DRAQ7 is a DNA fluorescent dye that only penetrates non-viable and permeabilized cells, allowing to evaluate transcellular permeability^36^. Visual examination of the stained tubules (Fig. 4A) revealed differences in cell permeabilization after exposure to the toxins, with those exposed to melittin (t = 24 h) showing the highest degree of DRAQ7 permeabilization in contrast to those exposed to the vehicle, followed by those exposed to ochratoxin A (t = 24 h). Exposure to neither nigericin (t = 5 h) nor patulin (t = 5 h) seemed to increase cell permeability to the dye, suggesting that although all toxins increase barrier permeability as shown by the TEER decrease observed, only the effect exerted by ochratoxin A and melittin was linked to an increase in transcellular permeability and cytotoxicity. To quantitatively assess this visual data, a CellProfiler image analysis pipeline was built, as explained in Fig. 4A, which allowed to identify DRAQ7-positive (DRAQ7 +) cells and the total number of cells per Caco-2 tubule. Analysis of the output data corroborated with what we observed visually, indicating that the percentage of DRAQ7 + cells increased in a dose-dependent manner after exposure to ochratoxin A and melittin (Fig. 4B,C). Draq7 permeability could not be investigated in all doses of patulin because tubule detachment was observed above from 3 µg/ml (Figure S5D).

### Nigericin disorganizes the actin cytoskeleton.

**Figure 5:**
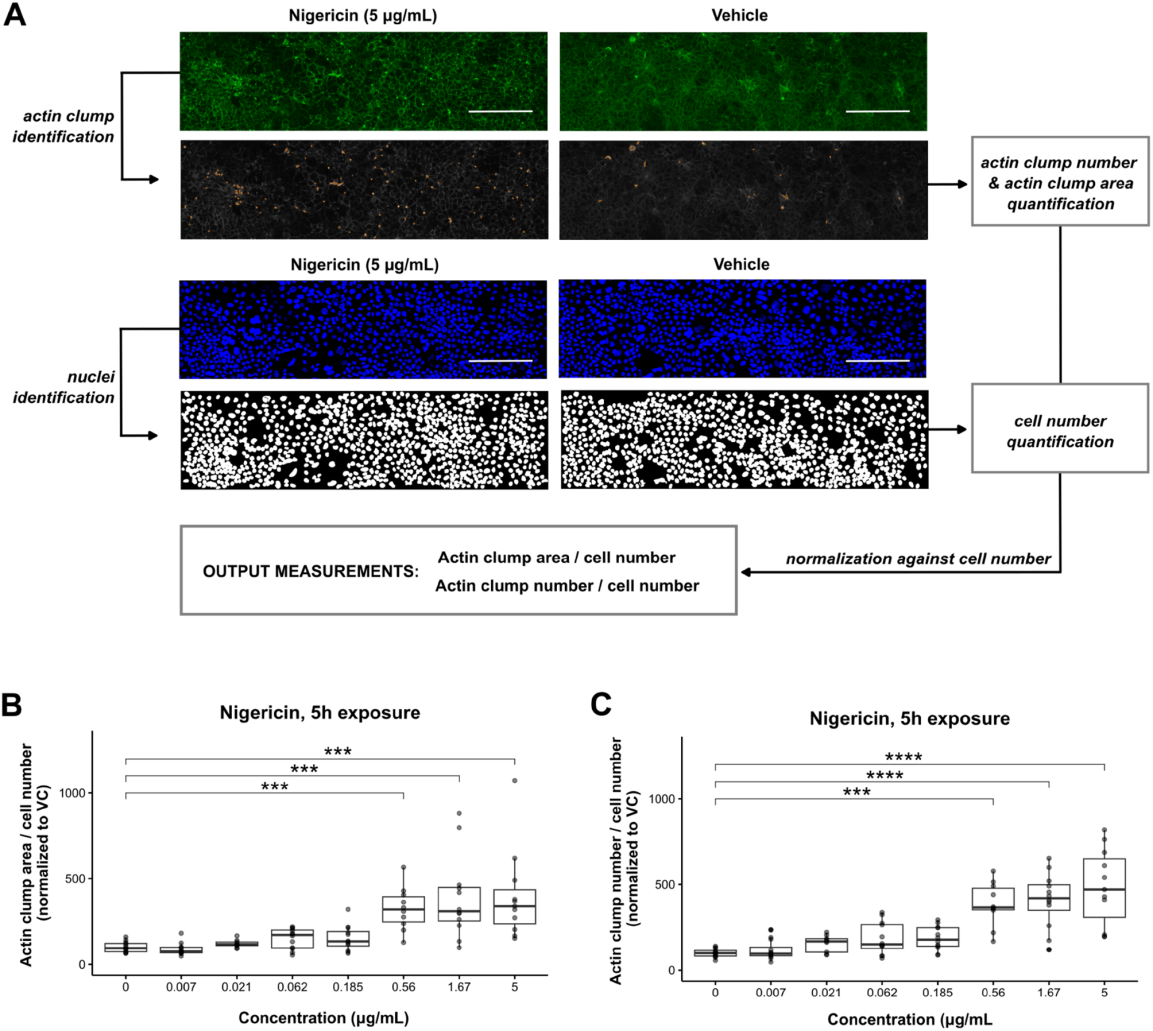
Quantification of nigericin-induced morphological changes in the actin cytoskeleton. Caco-2 tubules exposed to nigericin during 24 h were stained with ActinGreen 488 ReadyProbes Reagent. Nuclei were stained with NucBlue Fixed Cell ReadyProbes Reagent. (**A**) A CellProfiler pipeline was designed to identify actin clumps from confocal images and quantify actin clump number and area. Nuclei were also identified, which allowed quantification of cell number. Actin clump number and area were normalized against the cell number, obtaining the output measurements actin clump number/cell number and actin clump area/cell number, which were used as a measure of enterotoxin-induced actin disorganization and effect on cell morphology. Pictures are representative of three independent experiments (N = 3) with 2–4 technical replicates (n = 2–4) per enterotoxin concentration tested. Scale bar in white = 300 µm. (**B**,**C**) Dose–response increase in actin clump number (**B**) and actin clump area (**C**) after a 5 h exposure to nigericin. Three independent experiments (N = 3) were performed and 2–4 technical replicates (n = 2–4) were included per enterotoxin concentration tested. Significance was determined using Kruskal–Wallis test followed by Dunn’s post hoc test, where **** *p* < 0.0001; *** *p* < 0.001; ** *p* < 0.01; * *p* < 0.05.

To assess the effect of enterotoxin exposure on cellular morphology, we examined the changes in the actin cytoskeleton after exposure. Only exposure to nigericin (t = 5 h) produced observable changes in the actin cytoskeleton structure (Fig. 5A) in the shape of actin clump accumulation at the borders of the cell membrane, which were not observed after exposure to vehicle. Exposure to patulin (t = 5 h) did not produce such actin remodeling changes, pointing at actin remodeling as a nigericin-specific mechanism. To perform a dose–response quantification of this observed actin cytoskeleton disruption, a CellProfiler pipeline was designed to identify the number of accumulated actin clumps and calculate its occupied area, as well as quantifying the number of cells used to normalize the data, as indicated in Fig. 5A. Consistent with our previous visual observations, the mean actin clump area (Fig. 5B) and number (Fig. 5C) increased in a dose-dependent manner. This effect proved to be significantly different after exposure of the Caco-2 cells to nigericin concentrations higher than 0.56 µg/mL when compared against those treated with vehicle controls. Actin disorganization could not be investigated in Patulin above from 3 µg/ml because of tubule detachment (Figure S4B). Ochratoxin A and melittin did not induce dose-dependent actin reorganization (Figure S4C,D).”

### None of the enterotoxins induced NF-kB activation

Given the early involvement of the NF-kB signaling in pro-inflammatory responses, we evaluated NF-kB activation in the Caco-2 cells after exposure to all toxins for 1- or 3-h, using concentrations above the calculated EC50. NF-kB activation can be assessed visually by observing the translocation of the NF-kB p65 subunit in the nucleus. No detectable activation of NF-kB was observed. (Supplementary Figure S1).

## Discussion

In this study, we demonstrate that our Caco-2 tubular model is a promising multi-parametric and high-throughput platform for evaluation of the epithelial response to enterotoxins. We show that by combining different readouts, this platform allows to quantitatively assess toxin-specific alterations in barrier permeability, cytotoxicity, and cell morphology.

Each toxin elicited different responses as recorded by the readouts included in the model, reflecting their diverse pathogenic mechanism (Table 1). Melittin is the main component of honeybee venom, and due to its amphipathic properties can partition into the cell membrane, disrupting the lipid bilayer structure and possibly forming pores. This induces a fast permeabilization of water, ions and other molecules through the membrane and leads to rapid cell death^4,36^. Hence, melittin exerted a significant effect in cell membrane damage and cell permeabilization as measured by LDH release and DRAQ7 assays. Ochratoxin A and patulin can downregulate the expression of TJ proteins and induce apoptosis^6^. For ochratoxin A, DRAQ7 assays showed a significant decrease in cell viability, possibly reflecting the apoptotic effect of the toxin. Finally, while a decrease in barrier permeability was observed in TEER after exposure to Nigericin, this did not correlate with a loss of cell viability as measured by LDH release or DRAQ7, nor with breakage of the tubule.

**Table 1:** Summary of toxins’ signature.

	Incubation time (h)	TEER decrease (EC50) (µg/ml)	TEER decrease (hill slope)	DRAQ7 permeability	Actin remodeling	LDH increase
Nigericin	5	0.141	1.242	No	Yes	No
Patulin	5	6.469	4.827	No	No	Yes
Ochratoxin A	24	0.011	0.707	Yes (mild)	No	No
Melittin	24	13.55	26.047	Yes (high)	No	Yes

TEER is a widely used parameter to monitor barrier integrity in intestinal models, as it is a non-invasive technique that reflects changes in paracellular ionic conductivity dependent on cell shape, cell density, epithelial membrane properties and presence of TJs^37^. Here, the dose-dependent decrease observed in TEER after toxin exposure aligns with previous findings by studies using conventional *Transwell* models. However, in the OrganoPlate lower toxin concentrations of ochratoxin A and patulin were needed to induce such TEER decreases. In our case, 24 h exposures to ochratoxin A concentrations starting at 0.037 μg/mL (0.092 μM) resulted in 50% decrease in barrier function, whereas *Transwell* studies report 80% decreases following exposure to higher concentrations (2.5 μM and 100 μM)^38,39^. Similarly, whereas 5 h exposure to patulin showed TEER decrease at concentrations starting at 9.25 μg/mL (60 μM, 5 h) in the OrganoPlate, a *Transwell* study reports this effect at 100 µM (6 h incubation) in Caco-2^40^. This difference in toxin sensitivity was also observed by Pöschl et al*.* when examining the effect on TEER after exposure of the Caco-2 OrganoPlate model to mycotoxin deoxynivalenol^31^. We could attribute this to an increased enterotoxin uptake by the Caco-2 cells cultured in the OrganoPlate which would explain why we did not find higher sensitivity of our model to melittin when compared to published data on *Transwell*^41–43^. Indeed, intracellular entry of melittin is not required to physically damage the barrier^44^. For nigericin, we could not contrast our results because there were not any studies on its impact on the gut.

This higher toxin sensitivity observed in the OrganoPlate supports previous studies that report increased drug sensitivity in organ-on-a-chip models. Trietsch et al*.* compared the effect of the apoptotic agent staurosporine among Caco-2 cells cultured in the OrganoPlate and *Transwell* models, finding that this effect was only observed in the OrganoPlate^27^. Similarly, perfused liver and kidney organ-on-a-chip models also show enhanced drug sensitivity compared to static models^45^. This increased sensitivity is attributed to the shear-stress induced by dynamic medium flow conditions, which are not present in static *Transwell* models. Given that organ-on-a-chip models better mimic the structure and physiology of the intestine^26^, the increased drug sensitivity observed is likely to better reflect that of the human intestinal barrier. Seeing that most enterotoxin studies have so far relied on *Transwell* models for evaluating barrier integrity^46^, reconsideration of the effective doses is needed, especially regarding toxicological assessment studies on which public health food regulations may have been based on. Lastly, differences in medium composition among studies, including our use of serum-free medium, emphasize the need for careful consideration when comparing results.

DRAQ7 cytotoxicity staining assays have been described as a more sensitive, time-efficient, and non-toxic alternative to the gold-standard MTT assays, which often lead to false positive results due to reagent toxicity^47^. In our study, only melittin and ochratoxin A showed significant changes in cell permeability as measured by DRAQ7, correlating with their known cytotoxicity mechanisms. In addition to DRAQ7 staining, we also conducted LDH assays. These are a well-established method that determines cell viability by quantifying the levels of LDH enzyme present in the culture media after plasma membrane damage^47^. Combining both methods provides a comprehensive approach to studying cytotoxicity, with the LDH assay offering quantitative data and DRAQ7 staining providing visual confirmation of cell death. This dual approach enhances the reliability of cytotoxicity assessment, offering both quantitative measures and visual insights into the impact of toxins on cell viability. DRAQ7 proved to be more efficient in evaluating cell viability than LDH release assays, as the cytotoxic effect of ochratoxin A could only be detected with DRAQ7 staining and not by measuring LDH release. Moreover, the melittin concentration at which this cytotoxic effect was significant as measured by DRAQ7 (1.85 μg/mL) was lower than that determined by LDH release (5.56 μg/mL). This supports previous studies that suggest DRAQ7 staining is a more sensitive measurement of cell cytotoxicity^48^. However, as seen in the ochratoxin A study, certain enterotoxin doses may exert cell permeabilization effects without necessarily causing damage in the plasma membrane. Therefore, contrasting the results obtained from both assays leads to a more thorough characterization of the enterotoxin-specific effect on Caco-2 viability.

Since actin filaments are essential components of the TJs, and these complexes are targeted by ochratoxin A and patulin^6^, we were surprised to find that exposure of our model to these toxins did not lead to significant rearrangement of the actin cytoskeleton. However, we are the first to report that nigericin induces actin disorganization. This could be linked to the known effect of pH on actin dynamics, as nigericin’s ionophore activity induces cytoplasm acidification. Variations in pH have been shown to influence actin dynamics by modulating the activity of pH-sensing actin regulatory proteins^49^, and acidic pH has been found to decrease the depolymerization rate of actin filaments^50^. Since such effects in cell morphology were not visually apparent in the phase-contrast images of the tubules, actin staining needs to be considered as a readout to monitor masked enterotoxin effects on cell morphology. This is particularly relevant in enterotoxin studies, as certain toxins from enteropathogenic bacteria such as *Clostridium difficile* and *Bacillus cereus* can directly activate or inactivate actin-modulating enzymes, resulting in actin cytoskeleton remodeling^51–53^. Further investigations of the effect of such enterotoxins in the Caco-2 OrganoPlate model are needed to demonstrate how our platform effectively models enterotoxin-induced actin remodeling. It is important to note that actin disorganization could not be properly evaluated in patulin due to tubule detachment above 3 µg/ml. For ochratoxin A and melittin, we observed unspecific rearrangements also in vehicles. This is potentially due to the longer serum-deprivation (48 h), warranting additional investigations into the time-dependent dynamics of actin remodeling induced by these toxins.

Likewise, further investigation of the effects of enterotoxins on epithelial inflammation would heighten model validity and human translatability. Transcription factor NF-kB regulates epithelial pro-inflammatory cytokine expression^54^ and has been found to be constitutively activated in epithelial cells from patients with inflammatory bowel diseases^55^, making it a valuable readout to assess gut inflammation. Activation of NF-kB in intestinal epithelial cells requires receptor stimulation by pathogen and tissue damage molecules, including toxins such as *Bacteroides fragilis*’s enterotoxin, *Clostridium difficile*‘s toxin A and mycotoxin deoxynivalenol^56–58^. NF-kB activation was therefore expected after enterotoxin exposure. However, this effect was not observed after exposure to the toxins studied (Supplementary Figure S1). This suggests that the increase of barrier permeability is NF-kB -independent or that NF-kB activation occurred at an unrecorded timepoint or at different enterotoxin concentrations than the ones tested.

While the Caco-2 OrganoPlate model closely resembles the human intestinal epithelium, it does not fully recapitulate its cellular, tissue and organ complexity. Caco-2 cells exhibit differences in gene expression compared to human intestinal biopsies, which can be attributed to tumoral genetic abnormalities, as Caco-2 are a colorectal carcinoma-derived cell line^59^. Moreover, variability in culture conditions and heterogeneity of parental cell lines result in morphological and functional differences among different studies, potentially impacting the replicability of the findings^60^. No mucus, immune cells or commensal microorganisms are included in our model, which are mucosal components that can influence the enterotoxin effects in the epithelium^61^. However, the OrganoPlate platform allows successful integration of human intestinal organoids^62^, and co-culture of epithelial cells with mucus-producing and immune cells^63^. Assessing the effects of enterotoxin exposure in these more complex tissue environments would contribute to a more accurate modeling of human intestinal physiology in the OrganoPlate.

Altogether, our study highlights the potential of the OrganoPlate as a high-throughput, perfused, and membrane-free gut-on-a-chip platform to model the heterogeneous effects of enterotoxins on the intestinal epithelium. The enterotoxins tested in our study decreased barrier permeability by exerting different effects on cell morphology and cytotoxicity, which could be effectively measured and analyzed. This makes the Caco-2 OrganoPlate an attractive alternative to the conventional *Transwell* models and animal models that so far have governed enterotoxin research and food safety studies.

### Supplementary Information

Supplementary Figures.

## Data Availability

The datasets generated during this study are available from the corresponding authors on reasonable request.
