# Supplementary material for: A high-throughput gut-on-chip platform to study the epithelial responses to enterotoxins
Source: Sci Rep. 2024 Mar 9;14:5797. doi: 10.1038/s41598-024-56520-5 (PMC10925042; doi:10.1038/s41598-024-56520-5)
Supplement: Supplementary file 1 — Supplementary Figures. [file 41598_2024_56520_MOESM1_ESM.pdf]

Supplementary Material

Figure S1

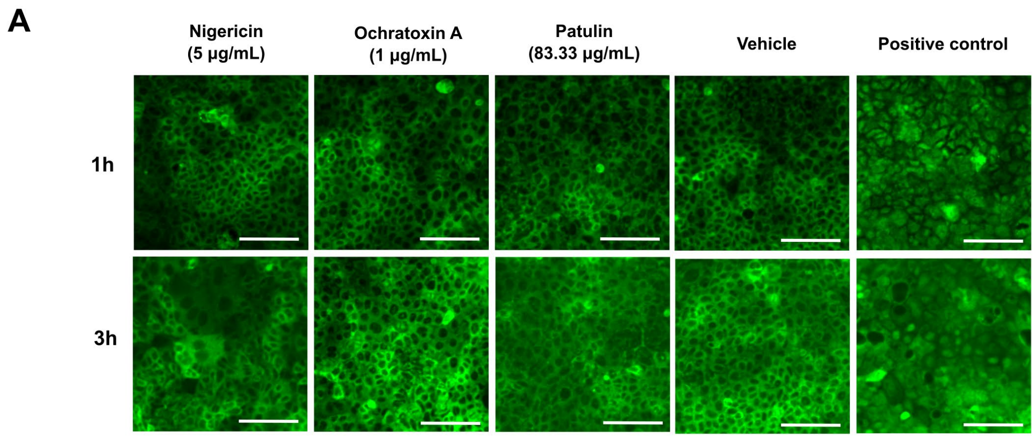

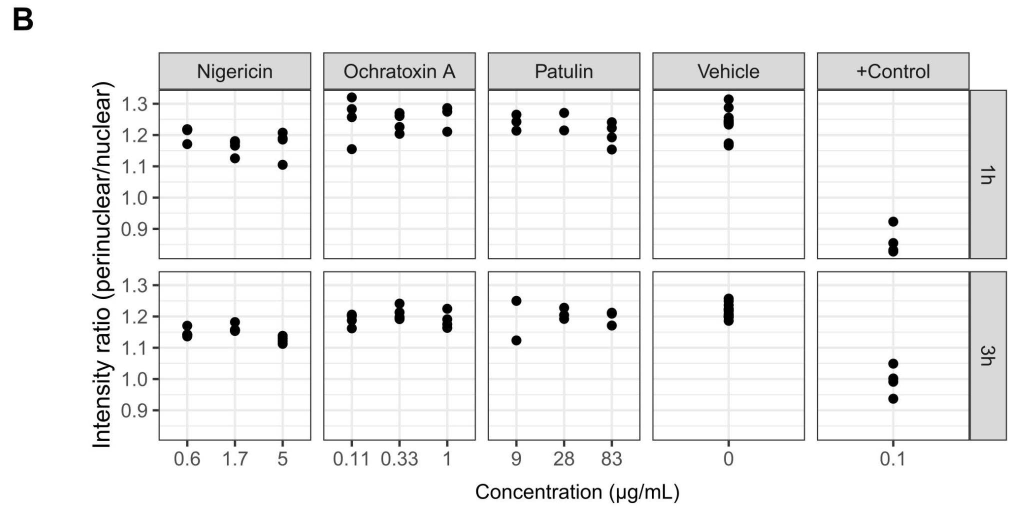

Supplementary Figure S1. **Lack of NF-kB Activation in Response to Toxin Exposure**. **A** NF-kB activation was assessed by staining the NF-kB p65 subunit after 1h and 3h exposure of Caco-2 tubules to nigericin, ochratoxin A, and patulin, with images representing the highest concentration tested. Melittin was excluded due to tubule detachment. Scale bar in white = 150 µm. **B** Quantification of p65 translocation into the nuclei for all concentrations tested. Data are from a single replicate (N =1, n = 2-4).

Figure S2

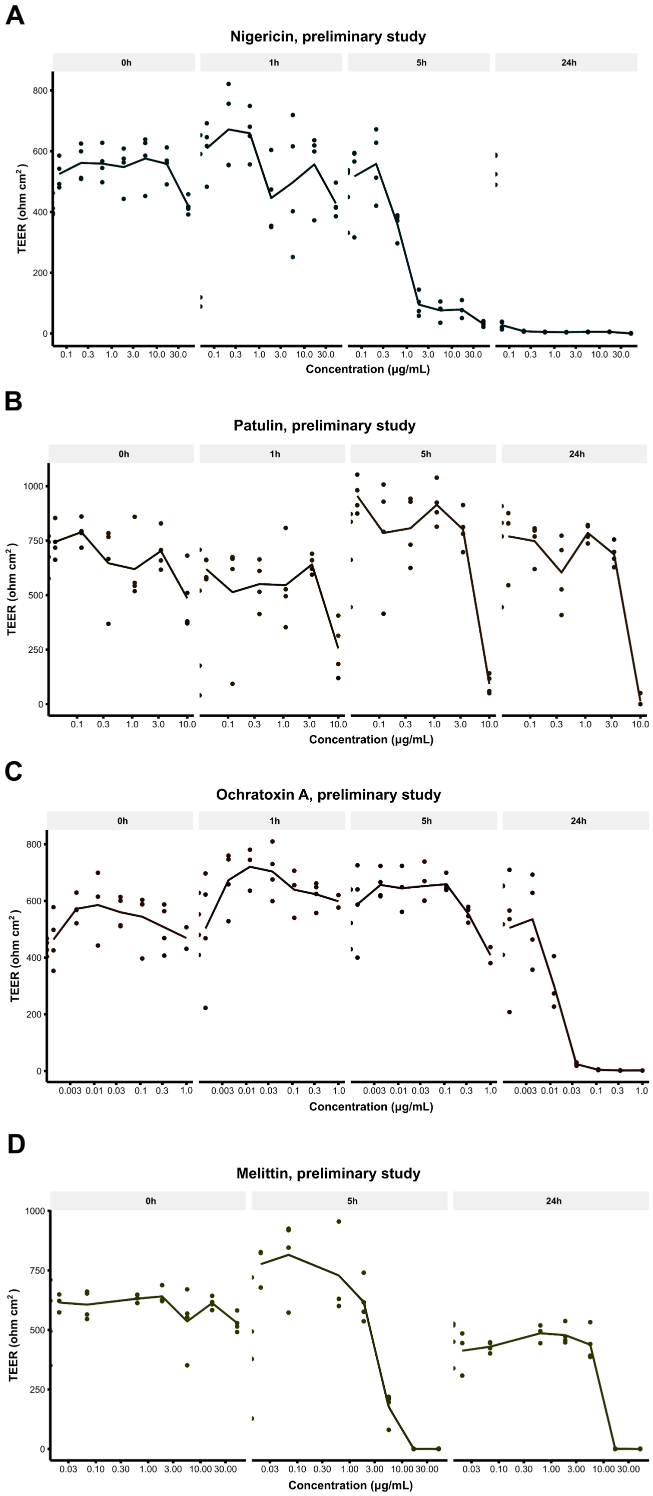

Supplementary Figure S2. Enterotoxin effect in TEER is time and concentration dependent. In a preliminary study, Caco-2 cells were incubated during 24h with various concentrations of nigericin (**A**), patulin (**B**), ochratoxin A (**C**) and melittin (**D**), and TEER was measured at the indicated timepoints. Raw TEER values are given in ohm*cm^2. Only data from a single replicate is shown (N =1, n = 2-4).

Figure S3

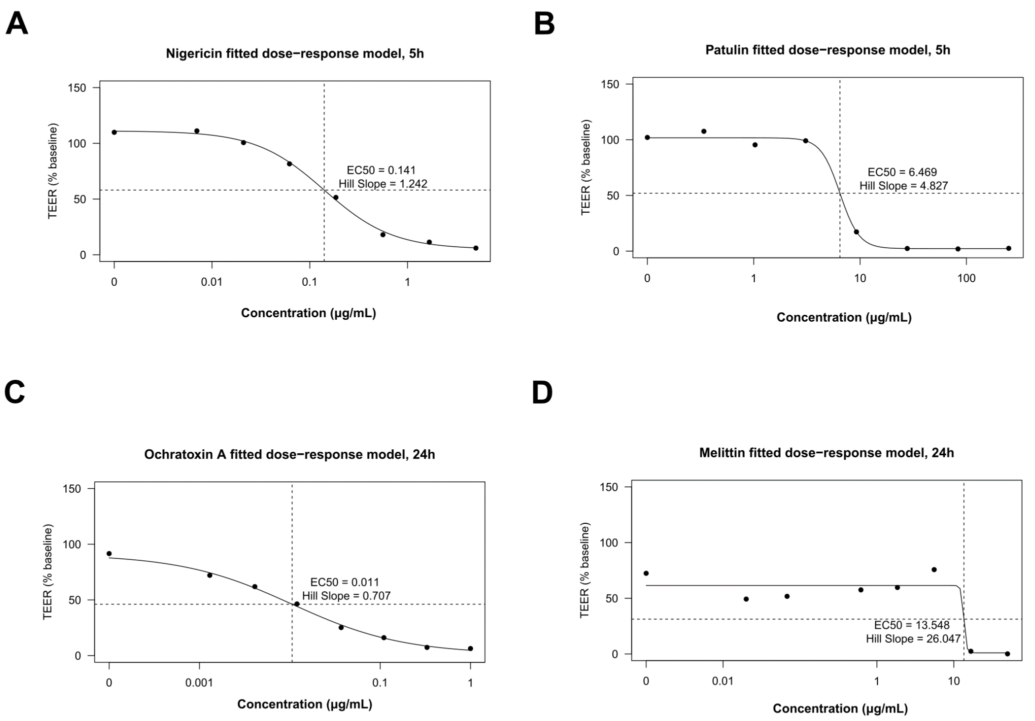

Supplementary Figure S3. Fitted dose-response regression curves allow the calculation of enterotoxin-specific EC50 values. Dose-response curves that result from fitting the dose-response TEER data normalized to baseline of nigericin (A), patulin (B), ochratoxin A (C) and melittin (D), with a dose-response regression curve. Enterotoxin concentrations are log transformed. EC50 values shown correspond to the toxin concentration at which TEER was decreased by 50%. Three independent experiments (N = 3) were performed and 2-4 technical replicates (n = 2-4) were included per enterotoxin concentration tested.

Figure S4

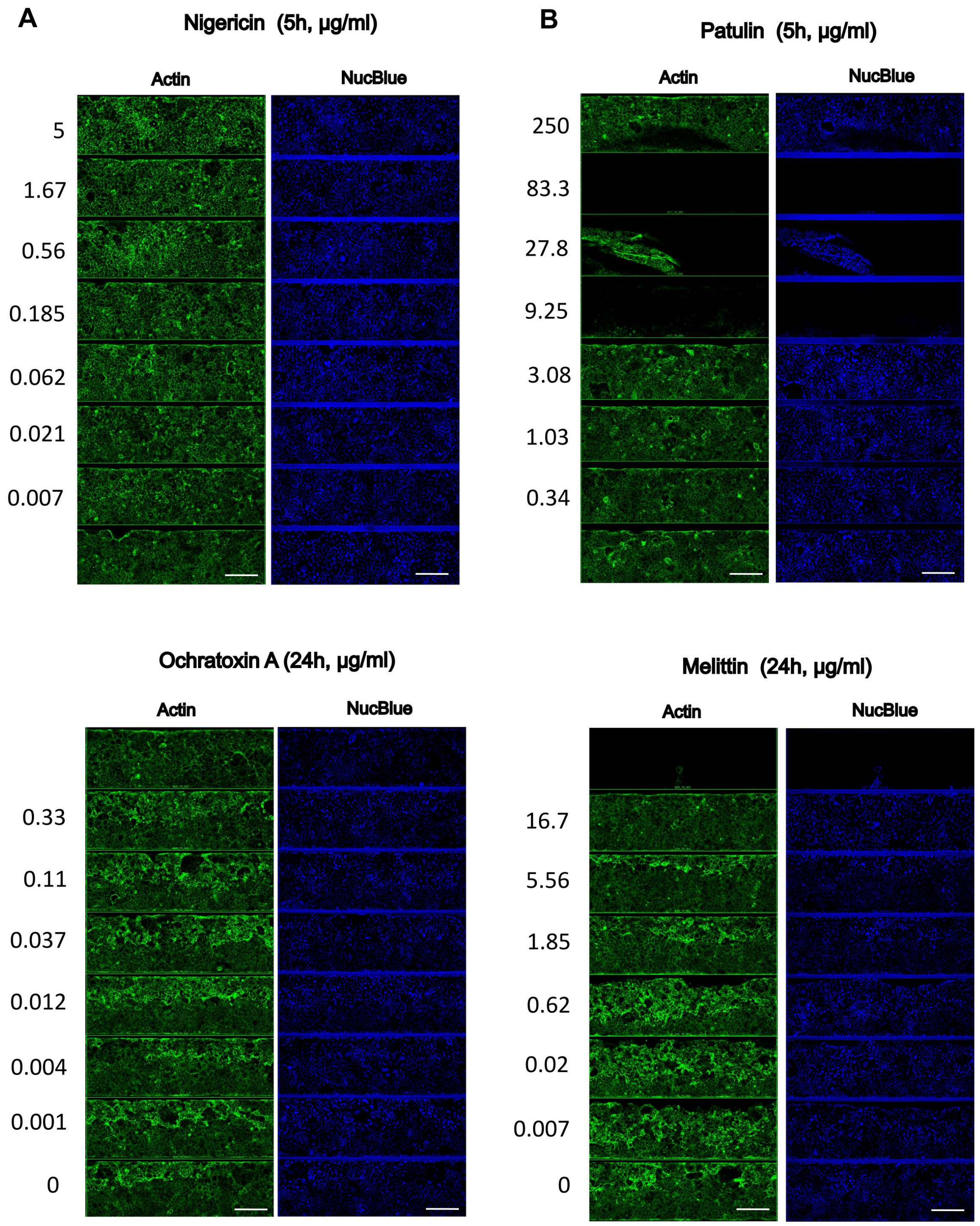

Supplementary Figure S4. Nigericin was the only enterotoxin to trigger dose-dependent actin disorganization. Caco-2 tubules exposed to Nigericin (A), Patulin (B), Ochratoxin A (C), and Melittin (D) were stained with ActinGreen 488 ReadyProbes Reagent. Nuclei were stained with NucBlue Fixed Cell ReadyProbes Reagent. Pictures are representative of three independent experiments (N = 3) with 2-4 biological replicates (n = 2-4) per enterotoxin concentration tested. Scale bar in white = 300µm

Figure S5

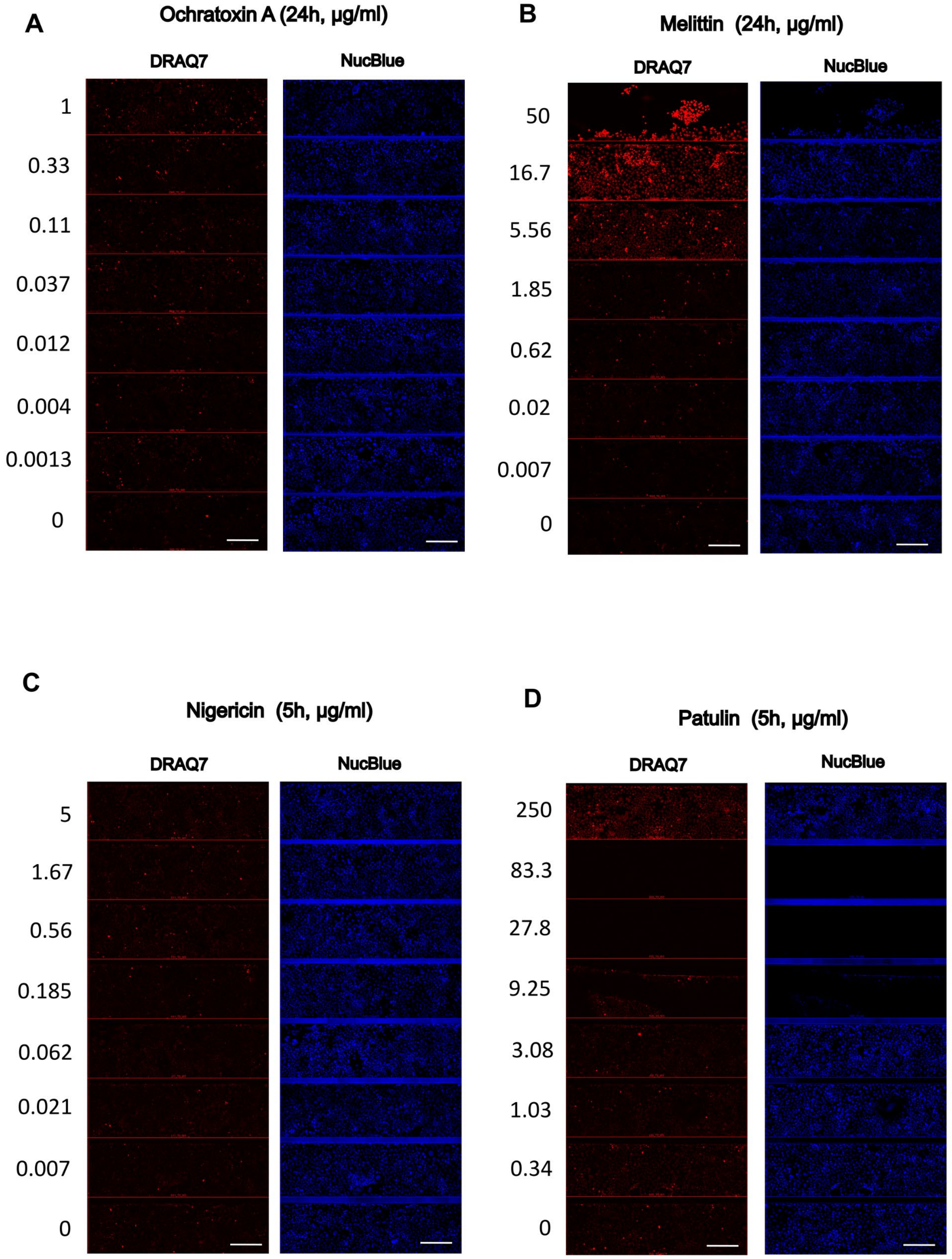

Supplementary Figure S5. DRAQ7 cell permeability was only observed after a 24h exposure to ochratoxin A and melittin. Caco-2 tubules exposed to ochratoxin A (**A**) and melittin (**B**), and nigericin (**C**) and patulin (**D**) during 5h were stained with DRAQ7 nuclear dye, which only penetrates permeabilized or dead cells. Nuclei were stained with NucBlue Fixed Cell ReadyProbes Reagent. Pictures are representative of three independent experiments (N = 3) with 2-4 biological replicates (n = 2-4) per enterotoxin concentration tested. Scale bar in white = 300µm.
